# Two New Quinazoline Derivatives from the Moss Endophytic Fungus *Aspergillus* sp. and Their Anti-inflammatory Activity

**DOI:** 10.1007/s13659-020-00287-5

**Published:** 2020-11-21

**Authors:** Ning-Ning Wang, Chun-Yu Liu, Tian Wang, Yue-Lan Li, Ke Xu, Hong-Xiang Lou

**Affiliations:** Department of Natural Product Chemistry, Key Lab of Chemical Biology of Ministry of Education, School of Pharmaceutical Sciences, Shandong University, Jinan, 250012 China

**Keywords:** Endophytic fungus, Quinazoline, Anti-inflammatory activity

## Abstract

**Abstract:**

Two new quinazoline derivatives versicomides E (**1**) and F (**2**), and 10 known compounds (**3**–**12**) were isolated from the moss endophytic fungus *Aspergillus* sp. Their structures were determined on the basis of extensive spectroscopic data analysis and ECD calculations. Among them, the compound **7** (6-hydroxy-3-methoxyviridicatin) was first reported as a natural product. Inhibition on LPS-induced NO production in RAW 264.7 murine macrophages found that compounds **5**, **7** and **8** showed significant inhibitory effects on NO production, with IC_50_ values of 49.85, 22.14 and 46.02 μM respectively.

**Graphic Abstract:**

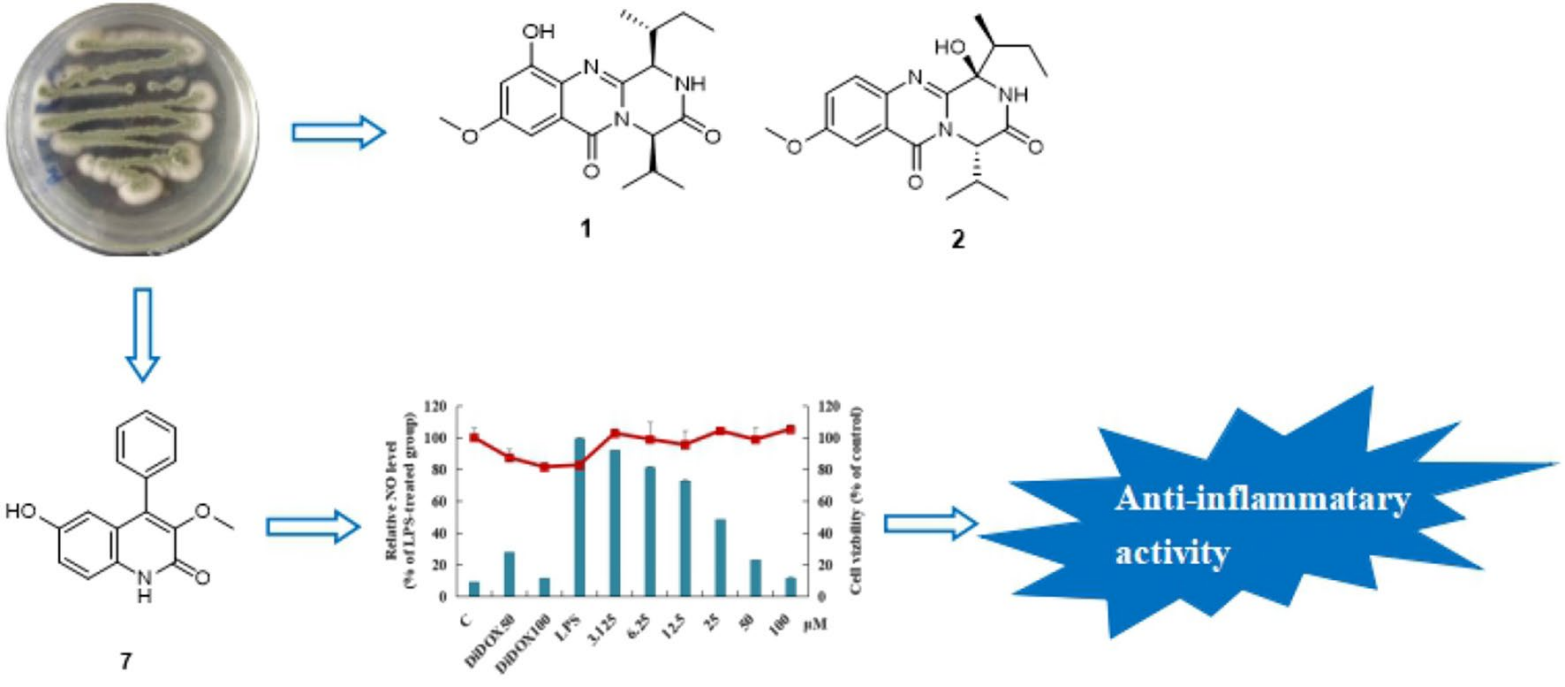

**Electronic supplementary material:**

The online version of this article (10.1007/s13659-020-00287-5) contains supplementary material, which is available to authorized users.

## Introduction

Endophytic fungi are defined as organisms that live in healthy internal tissues of plants without causing any immediate, obvious and negative effect [1, 2], and have been considered as useful and peerless sources of molecules with potent bioactivity [3], such as cytotoxic [4], antibacterial [5], antifungal [6], antioxidative [7], neuraminidase inhibitory [8] and phosphodiesterase inhibitory activities [9]. The moss, as the oldest branch of terrestrial plant evolution, has built extensive contact with fungi [10]. The moss endophytic fungus is also a great resource for bioactive compounds, reports of which were not as many as other plant endophytic fungi. Previously, there were some compounds including alkaloids, cyclic peptide, anthraquinone with antifungal activity [11], cytotoxic activity [12, 13], immunosuppressive activity [14] and allelopathy activity [15] that were discovered in moss endophytic fungi. In the present research, two new quinazoline derivatives [16] versicomides E (**1**) and F (**2**) and 10 known compounds (**3**–**12**) were obtained from the moss endophytic fungus *Aspergillus* sp. It is also the first report of the presence of compound **7** (6-hydroxy-3-methoxyviridicatin) as a natural product. Furthermore, anti-inflammatory assay with lipopolysaccharide (LPS)-activated RAW 264.7 murine macrophages found that compounds **5**, **7** and **8** showed strong inhibitory effects on NO production with IC_50_ values of 49.85, 22.14 and 46.02 μM respectively.

## Results and Discussion

**Fig. 1:**
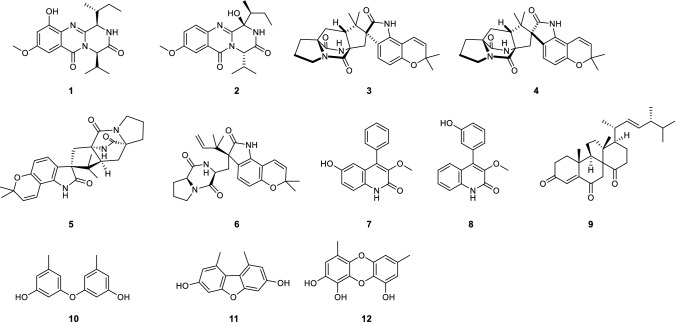
Structures of compounds **1**–**12**

**Fig. 2:**
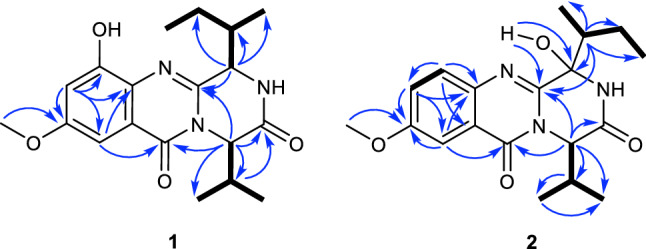
^1^H–^1^H COSY (H**—**H), key HMBC correlations (H → C) of compounds **1** and **2**

**Fig. 3:**
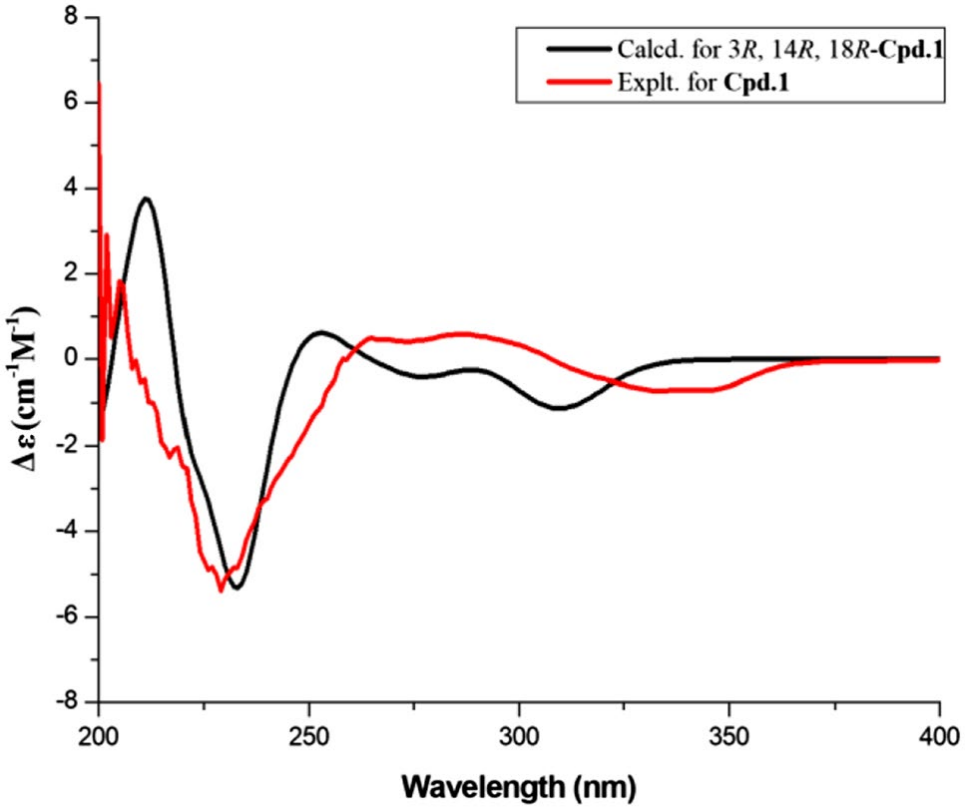
Calculated and experimental ECD spectra for compound **1**

Versicomide E (**1**) was isolated as faint yellow powder with the molecular formula C_19_H_25_N_3_O_4_ by means of HRESIMS (*m*/*z* 360.1919 [M + H]^+^, calcd. for 360.1923). The 1D NMR and HSQC spectra of compound **1** indicated that there was a methoxy (*δ*_H_ 3.82, *δ*_C_ 55.5, CH_3_-22) and a 1,3,4,5-tetrasubstituted benzene (*δ*_C_ 131.1, C-6; *δ*_C_ 154.1, C-7; *δ*_H_ 6.83, *δ*_C_ 107.7, C-8; *δ*_C_ 58.6, C-9; *δ*_H_ 6.99, *δ*_C_ 97.0, C-10; *δ*_C_ 120.9, C-11). Its ^13^C NMR and HMBC spectra confirmed the existence of a Val moiety and a similar Ile moiety, which was also validated by COSY. Then, compared with signals of quinazoline derivatives [16], compound **1** had similar chemical shifts with versicomide A. The difference between them was that versicomide A had 1,3,4-trisubstituted benzene ring while **1** had 1,3,4,5-tetrasubstituted with and a hydroxyl group (OH-7, *δ*_H_ 9.80, s) as revealed by ^1^H NMR. Therefore, the planar structure of **1** was elucidated as drawn in Fig. 1 which was also supported by the chemical shifts of C-7 (*δ*_C_ 154.1) and C-8 (*δ*_C_ 107.7). The absolute configuration was confirmed by chemical calculation of the ECD spectrum. According to the literature [16], we presumed the absolute configuration was the same as versicomide A. However, by comparing the experimental ECD data to the calculated result, the experimental ECD was consistent with the calculated ECD of (3*R*,14*R*,18*R*) (Figs. 2, 3). Then, the absolute configuration of compound **1** was determined. The optical rotation of [*α*]_D_^20^ =  − 88.5 (*c* 0.5, MeOH) also confirmed that.

Versicomide F (**2)** was also a faint yellow jelly. The molecular formula was determined to be C_19_H_25_N_3_O_4_ by HRESIMS (*m*/*z* 360.1919 [M + H]^+^, calcd. for 360.1923). Its ^1^H NMR showed that there was a different substitution from compound **1**, and a different type of hydroxyl group (OH-3, *δ*_H_ 6.58, s) from OH-7 (*δ*_H_ 9.80, s) in compound **1**. Compound **2** had 1,3,4-trisubstituted benzene ring, lacking a hydroxyl substituent was determined for the correlations from H-7 (7.64, d, 8.8 Hz) to C-6, C-8, C-9 and C-11, together with the COSY correlation of H-7 and H-8 in HMBC. HSQC indicated that C-3 (*δ*_C_ 84.9) was an oxygenated quaternary carbon. Combination with the HMBC correlations from OH-3 to C-3 and C-4, the planar structure of compound **2** was depicted as in Fig. 1. By comparing the ECD of **2** (Fig. S_20_) with that of the reported compound [16] leads to the final determination of the absolute configuration of compound **2** as 3*R*,14*S*,18*S*.

Compound **7** was reported as a natural product for the first time in this article. The structure of **7** was determined by comparing spectroscopic data with 3,6-*O*-dimethylviridicatin in literature [17] and determined as 6-hydroxy-3-methoxyviridicatin.

Other compounds were identified as versicolamide B (**3**) [18], taichunamide E (**4**) [19], notoamide B (**5**) [20], notoamide C (**6**) [21], 3-*O*-methylviridicatol (**8**) [17], dankasterone B (**9**) [22], diorcinol (**10**) [23], 3,7-dihydroxy-1,9-dimethyldibenzofuran (**11**) [24], aspergilol E (**12**) [25] by direct comparison of their spectral data with the reported.

**Fig. 4:**
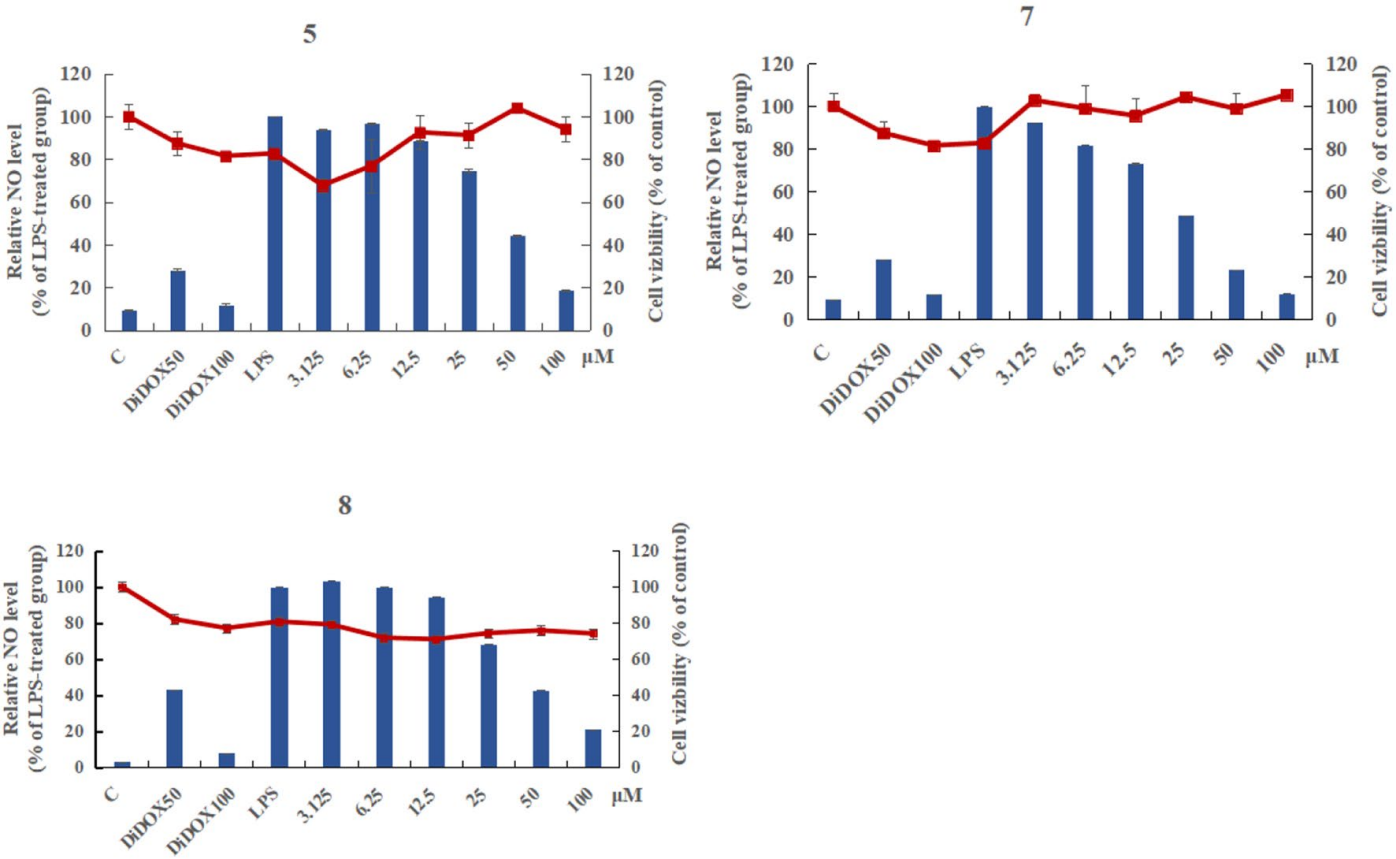
Inhibitory activity of compounds **5**, **7** and **8** against NO production in RAW 264.7 cells. Cells were treated with various concentrations of compounds along with LPS (1 μg/mL) for 24 h, and the accumulation of nitrite was evaluated by Griess reagent. Values were presented as mean ± SD from three independent experiments. *Column* relative NO level, *Dot* cell viability, *C* control, *DiDOX* positive control

All compounds were evaluated for the anti-inflammatory activity with the model to inhibit NO production in LPS-stimulated RAW 264.7 murine macrophages. Compounds **5**, **7** and **8** displayed strong inhibitory effects on NO production, with IC_50_ values of 49.85, 22.14 and 46.02 μM (Fig. 4) respectively.

## Experimental

### General Experimental Procedures

Optical rotations were acquired using a PerkinElmer 241MC polarimeter (Anton Paar GmbH, Graz, Austria) at 20 °C. UV data were obtained by a UV-2450 spectrophotometer (Shimadzu, Japan). CD spectra were performed on a Chirascan spectropolarimeter (Applied Photophysics Ltd., Leatherhead, UK). NMR spectra were recorded on a Bruker Avance spectrometer operating at 400 (^1^H) and 100 (^13^C) MHz or at 600 (^1^H) and 150 (^13^C) MHz with tetramethylsilane as an internal standard. HRESIMS data were determined by using a Finnigan LC-QDECA mass spectrometer. IR spectra were recorded on a Nicolet iN 10 Micro FTIR spectrometer. HPLC were performed on an Agilent 1200 G1311A pump equipped with a G1322A degasser, a G1315D DAD detector, and an Eclipse XDB-C_18_ 5 μm column (9.4 × 250 mm). Column chromatography (CC) was carried out using silica gel (200–300 mesh; Qingdao Haiyang Chemical Co. Ltd., Qingdao, China) and Sephadex LH-20 (25–100 mm; Pharmacia Biotek, Denmark). TLC was carried out with precoated silica gel GF-254 glass plates (Qingdao Haiyang Chemical Co. Ltd., Qingdao, China). The compounds were visualized under UV (254 nm) light and by spraying with anisaldehyde–H_2_SO_4_ followed by heating.

### Fungal Material

The fungus *Aspergillus* sp. was isolated from the *Trichocoleaceae* sp., collected from Wuyanling Nature Reserve Area, Zhejiang Province, China. The strain (No. 7-2-1) was identified using nuclear ITS rDNA sequences (GenBank MN310533.1), and was deposited in the Key Lab of Chemical Biology of Ministry of Education, Shandong University, Jinan, China.

The fungus was cultured in eight 300 mL Erlenmeyer flasks, each containing 100 mL of potato dextrose broth (PDB), and put them on a rotary shaker (120 rpm) at 25 °C for 7 days. Large-scale fermentation was conducted in 60 Erlenmeyer flasks (500 mL), containing 80 g of rice and 120 mL of distilled H_2_O. Then autoclave at 120 °C for 30 min, and after cooling to room temperature, each flask was inoculated with 10 mL seed culture and incubated under static condition at room temperature for 50 days.

### Extraction and Isolation

The fermented material including the mycelium was extracted with EtOAc for three times, and the organic solvent was evaporated under vacuum to yield the crude extract (108 g). The crude extract was separated into 15 fractions (A–O) using a silica gel column with a step gradient of CH_2_Cl_2_/MeOH from 100:0 to 0:100 (v/v).

Fr. D (4.9 g) was chromatographed by a silica gel column with step gradient of PE/EtOAc from 100:0 to 0:100 (v/v) to yield 15 subfractions (D_A_–D_N_). Fr. D_M_ (16.2 mg) was purified by HPLC (80% MeOH/H_2_O, 1.5 mL) to yield compound **6** (2.4 mg, *t*_R_ = 14.0 min). Fr. D_N_ (304.7 mg) and Fr. D_H_ (810.0 mg) were both isolated with Sephadex LH-20 CC eluted with CH_2_Cl_2_/MeOH (1:2 and 1:1) to afford 8 parts (D_N1_–D_N8_ and D_H1_–D_H8_). Fr. D_N3_ (14.4 mg) was prepared with HPLC (72% MeOH/H_2_O, 1.5 mL) to yield compound **10** (1.0 mg, *t*_R_ = 13.0 min). Fr. D_H2_ (385 mg) was separated into 17 parts (D_H2a_–D_H2q_) using MPLC (ODS, MeOH/H_2_O from 10:90 to 100:0). Further purification of Fr. D_H2f_ (15.0 mg) and Fr. D_H2m_ (18.3 mg) with HPLC gave compound **1** (40% MeCN/H_2_O, 1.5 mL, 2.8 mg, *t*_R_ = 12.2 min), **2** (40% MeCN/H_2_O, 1.5 mL, 6.2 mg, *t*_R_ = 10.0 min) and **9** (80% MeCN/H_2_O, 1.5 mL, 6.4 mg, *t*_R_ = 14.0 min). Compound **11** (2.7 mg, *t*_R_ = 20.5 min) was obtained by purifying Fr. D_H7_ (60.7 mg) with HPLC (62% MeOH/H_2_O, 1.5 mL).

Fr. G (8.7 g) was chromatographed by a silica gel column with step gradient of PE/EtOAc from 100:0 to 0:100 (v/v) to yield 23 subfractions (G_A_–G_w_). Fr. G_M_ (3.0 g) was first fractionated by Sephadex LH-20 CC eluted with CH_2_Cl_2/_MeOH (1:1) into seven subfractions (G_M1_–G_M7_). Fr. G_M5_ (3.0 g) was isolated into twelve parts (G_M5a_–G_M5l_) by MPLC (ODS, MeOH/H_2_O from 10:90 to 100:0), and then, Fr. G_M5j_ (69.9 mg) was subjected to Sephadex LH-20 CC by elution with MeOH to obtain six parts (G_M5j1_–G_M5j6_). Preparation of Fr. G_M5j5_ (49.5 mg) with HPLC (77% MeOH/H_2_O, 1.5 mL) gave compound **12** (2.0 mg, *t*_R_ = 4.2 min).

Fr. I (553.5 mg) was first fractionated using Sephadex LH-20 CC with the mobile phase of CH_2_Cl_2_/MeOH (2:1), and 11 subfractions (I_1_–I_11_) were obtained. Fr. I_2_ (69.0 mg) was purified by HPLC (45% MeCN/H_2_O, 1.5 mL) to afford compound **3** (15.8 mg, *t*_R_ = 15.0 min), **4** (5.7 mg, *t*_R_ = 16.0 min), and **5** (3.9 mg, *t*_R_ = 20.0 min). Fr. I_5_ (53.8 mg) was isolated using Sephadex LH-20 CC eluted with MeOH, and got 7 subfractions (I_51_–I_57_). Purification of Fr. I_53_ (42.7 mg) by HPLC (54% MeOH/H_2_O, 1.5 mL) gave compound **7** (2.4 mg, *t*_R_ = 17.0 min) and **8** (2.6 mg, *t*_R_ = 23.8 min).

### Spectroscopic Data of Compounds

#### Versicomide E (**1**)

Faint yellow powder; [*α*]_D_^20^ =  − 88.5 (*c* 0.5, MeOH); UV (MeOH) λ_max_ (log *ε*) 209 (2.66), 242 (3.11) nm; IR (KBr) *ν*_max_ 1662, 1629, 1590, 1467, 1383 cm^−1^; ECD (MeOH): 200 (Δ*ε* + 3.49), 229 (Δ*ε − *5.13) nm; ^1^H and ^13^C NMR data: Table 1; HRESIMS *m*/*z* 360.1919 for [M + H]^+^ (calcd 360.1923 for C_19_H_25_N_3_O_4_).

**Table 1:** ^1^H and ^13^C NMR spectral data for compounds **1** and **2** in DMSO-*d*_6_

Position	**1**		**2**	
	*δ*_H_^a^ (*J* in Hz)	*δ*_C_^b^	*δ*_H_^c^ (*J* in Hz)	*δ*_C_^d^
1		167.6, C		169.8, C
2	8.40, s		8.68, s	
3	4.68, d (1.8)	58.0, CH		84.9, C
3-OH			6.58, s	
4		147.3, C		150.1, C
5				
6		131.1, C		141.1, C
7		154.1, C	7.64, d (8.8)	129.6, CH
7-OH	9.80, s			
8	6.83, d (2.7)	107.7, CH	7.46, d (8.8)	125.0, CH
9		158.6, C		158.7, C
10	6.99, d (2.7)	97.0, CH		106.8, C
11		120.9, C		121.1, C
12		160.2, C		161.1, C
13				
14	4.91, d (8.7)	60.4, CH	4.90, d (8.8)	60.7, CH
15	2.24, m	30.4, CH	2.62, m	32.9, CH
16	0.83, d (6.7)	19.8, CH_3_	1.10, d (6.4)	20.7, CH_3_
17	1.03, d (6.7)	19.1, CH_3_	0.79, d (6.4)	19.7, CH_3_
18	2.93, m	34.7, CH	2.71, m	41.6, CH
19	1.13, d (7.3)	15.4, CH_3_	1.13, d (6.4)	11.4, CH_3_
20	1.24, m	22.7, CH_2_	1.12, m 1.20, m	25.0, CH_2_
21	0.83, t (7.2)	12.4, CH_3_	0.84, t (7.1)	13.1, CH_3_
22	3.82, s	55.5, CH_3_	3.89, s	56.2, CH_3_

^a^Measured at 600 MHz

^b^Measured at 150 MHz

^c^Measured at 400 MHz

^d^Measured at 100 MHz

#### Versicomide F (**2**)

Faint yellow jelly; [*α*]_D_^20^ =  − 32.4 (*c* 1.0, MeOH); UV (MeOH) λ_max_ (log *ε*) 226 (2.62), 279 (0.95) nm; IR (KBr) *ν*_max_ 1677, 1617, 1597, 1492, 1361 cm^−1^; ECD (MeOH): 219 (Δ*ε* + 2.59), 238 (Δ*ε* − 4.83) nm; ^1^H NMR and ^13^C NMR data: Table 1; HRESIMS *m*/*z* 360.1919 for [M + H]^+^ (calcd 360.1923 for C_19_H_25_N_3_O_4_).

### Anti-inflammatory Activity Test

The inhibition of NO production assay was performed according to the reported procedures [26]. DiDOX was set up as a positive control group. Then the percentage of NO production inhibition was calculated to get the IC_50_ values.

## Electronic supplementary material

(DOCX 6850 kb)
