# Supplementary material for: Two New Quinazoline Derivatives from the Moss Endophytic Fungus Aspergillus sp. and Their Anti-inflammatory Activity
Source: Nat Prod Bioprospect. 2020 Nov 21;11(1):105–10. doi: 10.1007/s13659-020-00287-5 (PMC7933300; doi:10.1007/s13659-020-00287-5)
Supplement: Supplementary file 1 — (DOCX 6850 kb) [file 13659_2020_287_MOESM1_ESM.docx]

**Supplementary data for**

# Two New Quinazoline Derivatives from the Moss Endophytic Fungus *Aspergillus* sp. and Their Anti-inflammatory Activity

Ning-Ning Wang^1^ · Chun-Yu Liu^1^ · Tian Wang^1^ · Yue-Lan Li^1^ · Ke Xu^1^ · Hong-Xiang Lou^1^

Ning-Ning Wang and Chun-Yu Liu have contributed equally to this work.

*Department of Natural Product Chemistry, Key Lab of Chemical Biology of Ministry of Education, School of Pharmaceutical Sciences,* *Shandong University, Jinan 250012, China*

* Corresponding Author

Hong-Xiang Lou

louhongxiang@sdu.edu.cn

**Contents**

[Figure S_1_. ^1^H NMR spectrum (600 MHz) of 1 in DMSO-d6. 3](#_Toc15569)

[Figure S_2_. ^13^C NMR spectrum (150 MHz) of 1 in DMSO-d6. 3](#_Toc32229)

[Figure S_3_. HSQC spectrum (600 MHz) of 1 in DMSO-d6. 4](#_Toc24621)

[Figure S_4_. HMBC spectrum (600 MHz) of 1 in DMSO-d6. 4](#_Toc21373)

[Figure S_5_. ^1^H-^1^H COSY spectrum (600 MHz) of 1 in DMSO-d6. 5](#_Toc19206)

[Figure S_6_. HRESIMS spectrum of 1. 5](#_Toc20526)

[Figure S_7_. UV spectrum of 1. 6](#_Toc7395)

[Figure S_8_. CD spectrum of 1. 6](#_Toc1006)

[Figure S_9_. IR spectrum of 1. 7](#_Toc574)

[Figure S_10_. ^1^H NMR spectrum (400 MHz) of 2 in DMSO-d6. 7](#_Toc23591)

[Figure S_11_. ^13^C NMR spectrum (100 MHz) of 2 in DMSO-d6. 8](#_Toc16975)

[Figure S_12_. HSQC spectrum (400 MHz) of 2 in DMSO-d6. 8](#_Toc7392)

[Figure S_13_. HMBC spectrum (400 MHz) of 2 in DMSO-d6. 9](#_Toc27650)

[Figure S_14_. ^1^H-^1^H COSY spectrum (400 MHz) of 2 in DMSO-d6. 9](#_Toc4171)

[Figure S_15_. NOESY spectrum (400 MHz) of 2 in DMSO -d6. 10](#_Toc9173)

[Figure S_16_. HRESIMS spectrum of 2. 10](#_Toc1977)

[Figure S_17_. UV spectrum of 2. 11](#_Toc24946)

[Figure S_18_. CD spectrum of 2. 11](#_Toc24902)

[Figure S_19_. IR spectrum of 2. 12](#_Toc9020)

[Figure S_20_. Experimental ECD spectra of 2. 12](#_Toc9020)

##### Figure S_1_. ^1^H NMR spectrum (600 MHz) of 1 in DMSO-d6.

##### Figure S_2_. ^13^C NMR spectrum (150 MHz) of 1 in DMSO-d6.

##### Figure S_3_. HSQC spectrum (600 MHz) of 1 in DMSO-d6.

##### Figure S_4_. HMBC spectrum (600 MHz) of 1 in DMSO-d6.

##### Figure S_5_. ^1^H-^1^H COSY spectrum (600 MHz) of 1 in DMSO-d6.


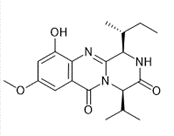

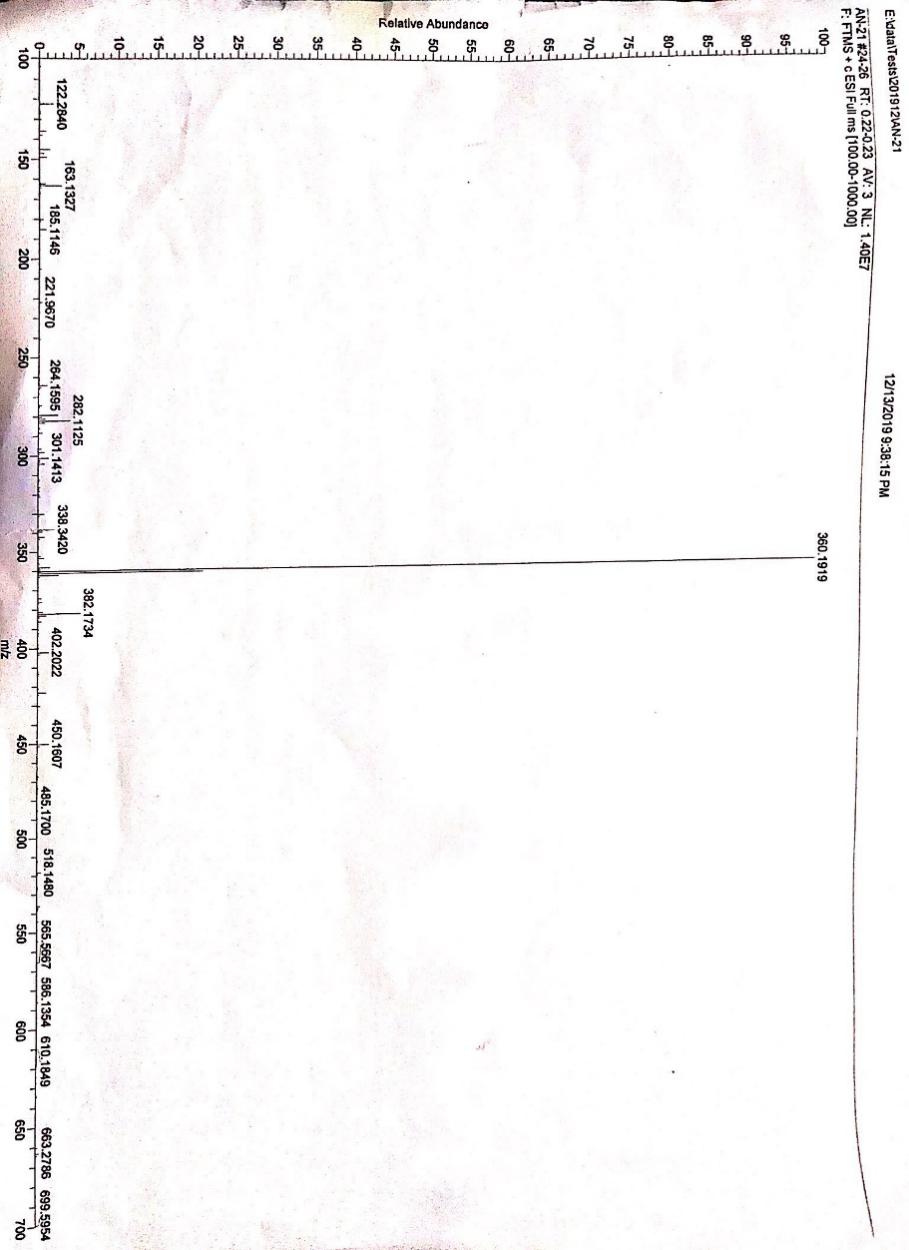


##### Figure S_6_. HRESIMS spectrum of 1.


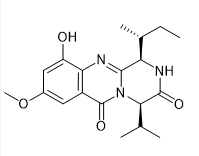

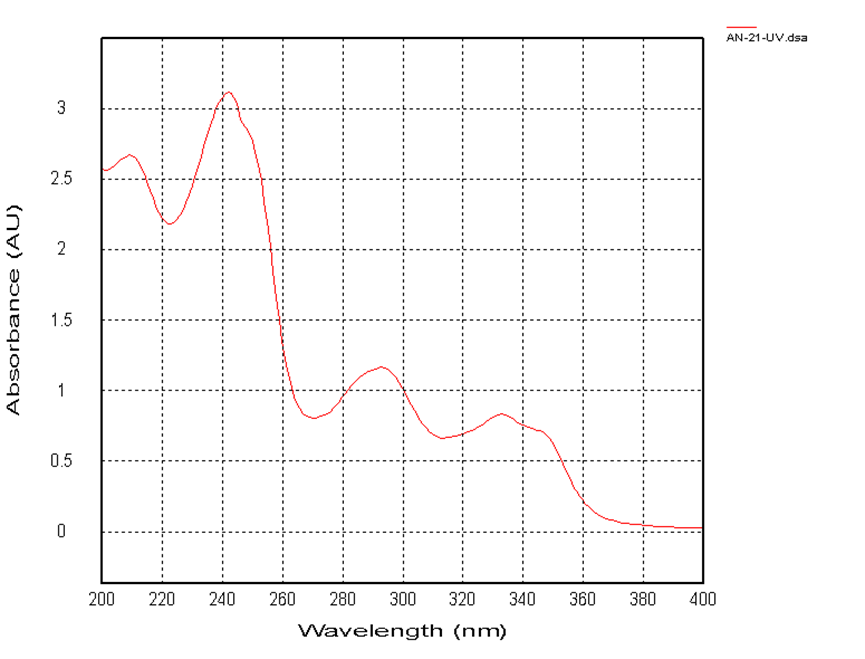


Figure S_7_. UV spectrum of 1.


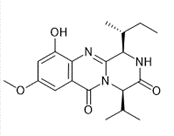

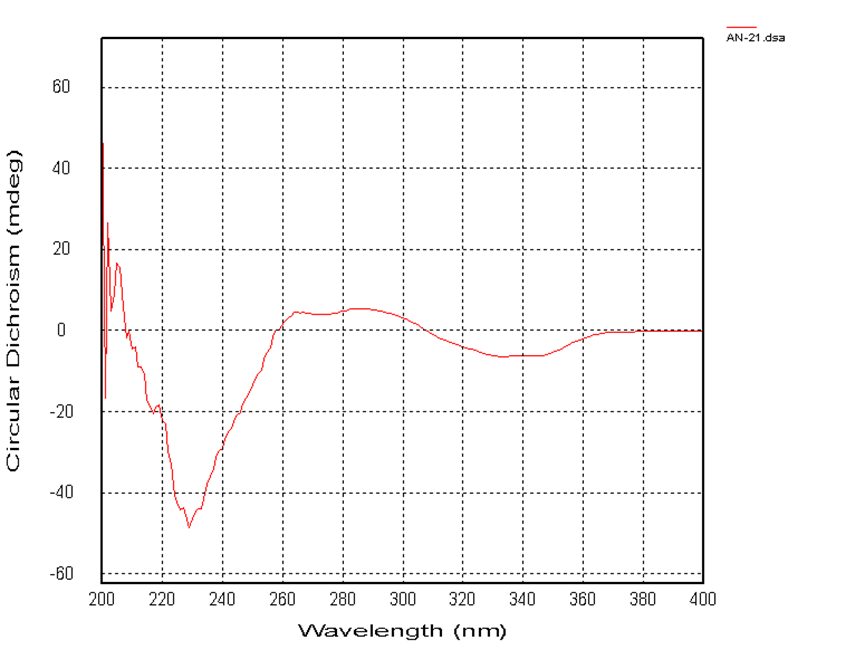


##### Figure S_8_. CD spectrum of 1.


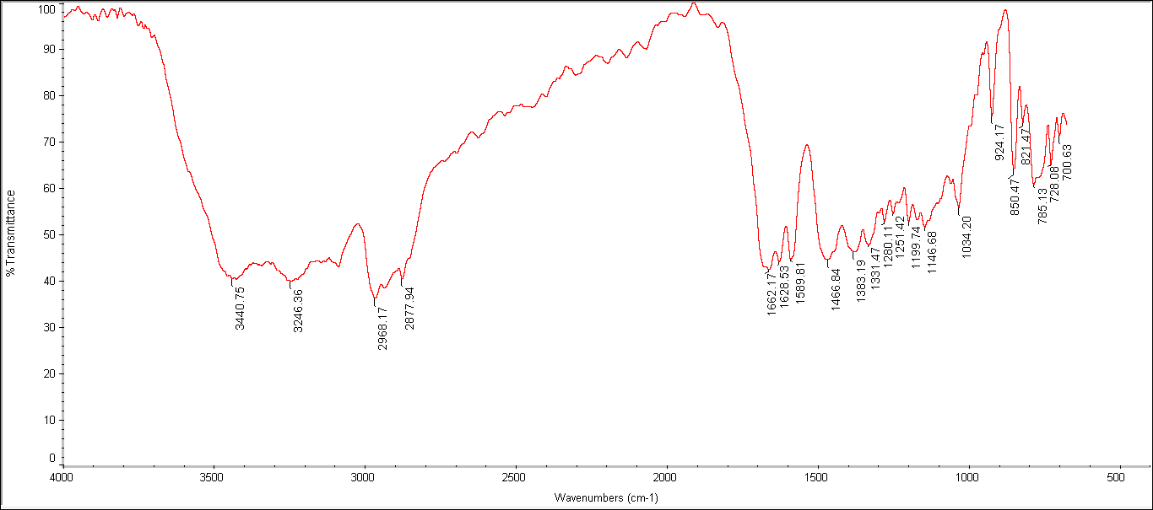


##### Figure S_9_. IR spectrum of 1.

##### Figure S_10_. ^1^H NMR spectrum (400 MHz) of 2 in DMSO-d6.

##### Figure S_11_. ^13^C NMR spectrum (100 MHz) of 2 in DMSO-d6.

##### Figure S_12_. HSQC spectrum (400 MHz) of 2 in DMSO-d6.

##### Figure S_13_. HMBC spectrum (400 MHz) of 2 in DMSO-d6.

##### Figure S_14_. ^1^H-^1^H COSY spectrum (400 MHz) of 2 in DMSO-d6.

##### Figure S_15_. NOESY spectrum (400 MHz) of 2 in DMSO -d6.


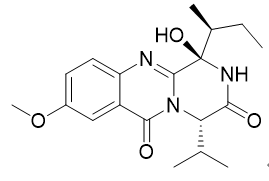

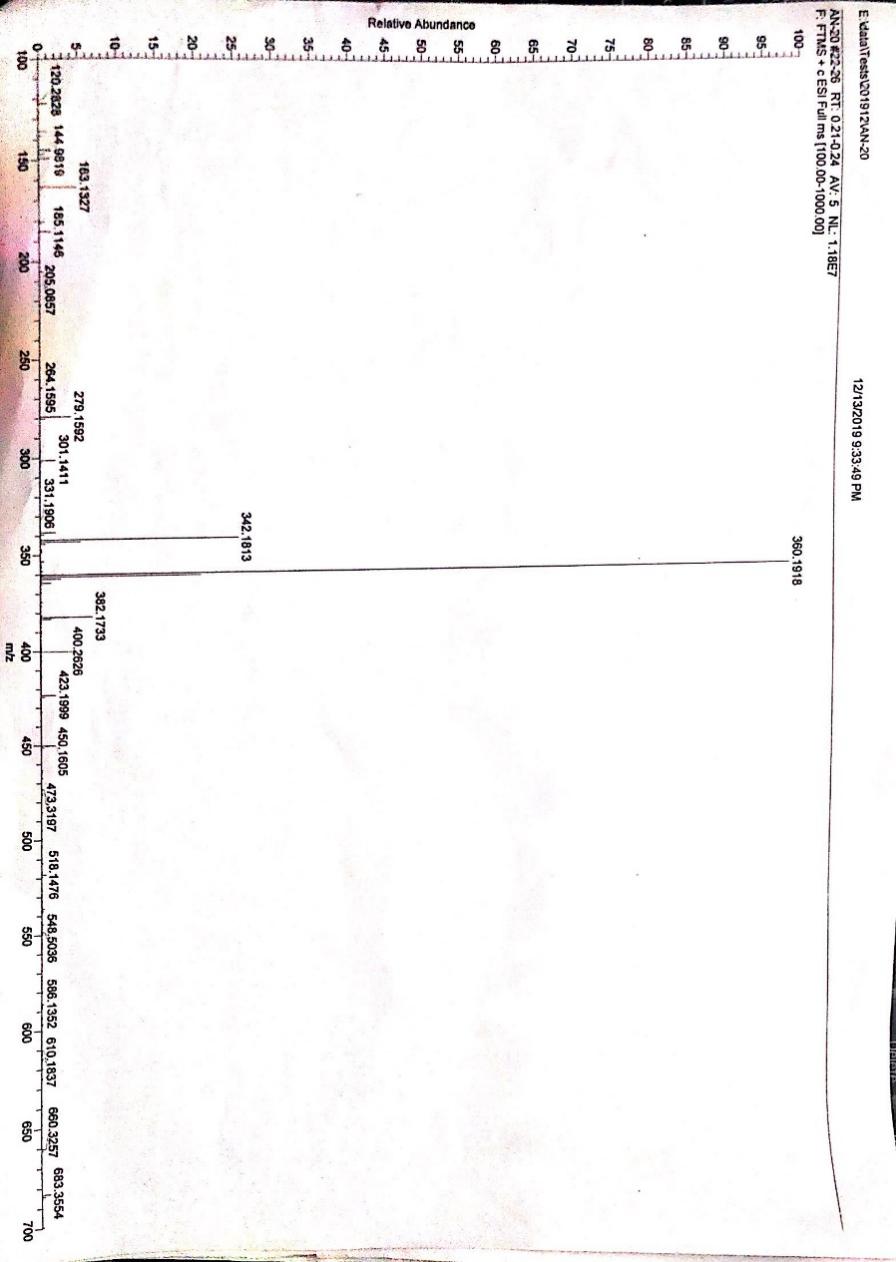


##### Figure S_16_. HRESIMS spectrum of 2.


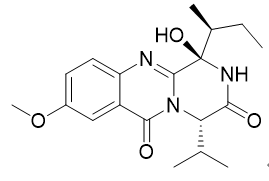

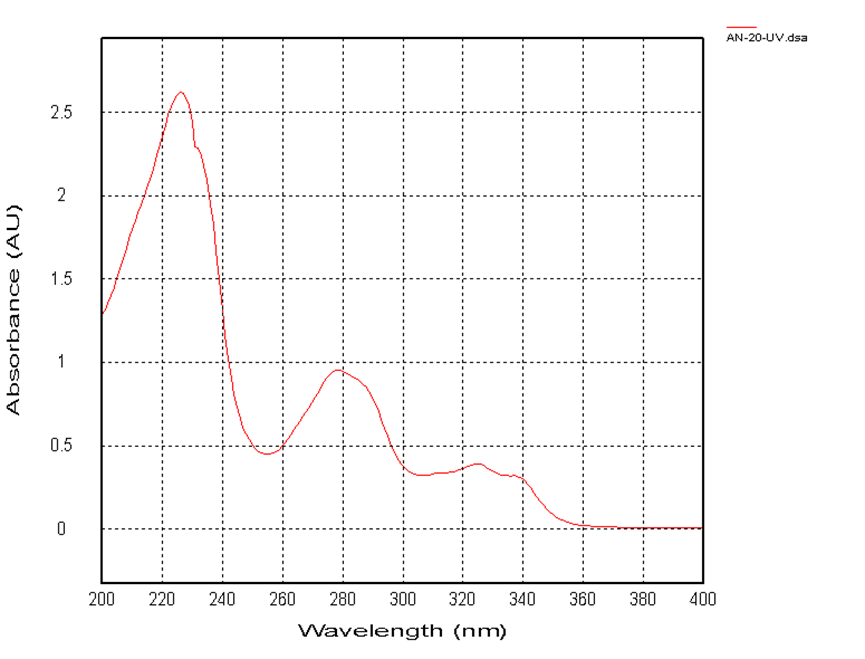


##### Figure S_17_. UV spectrum of 2.


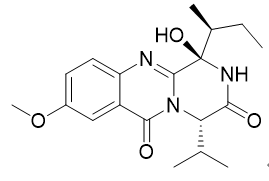

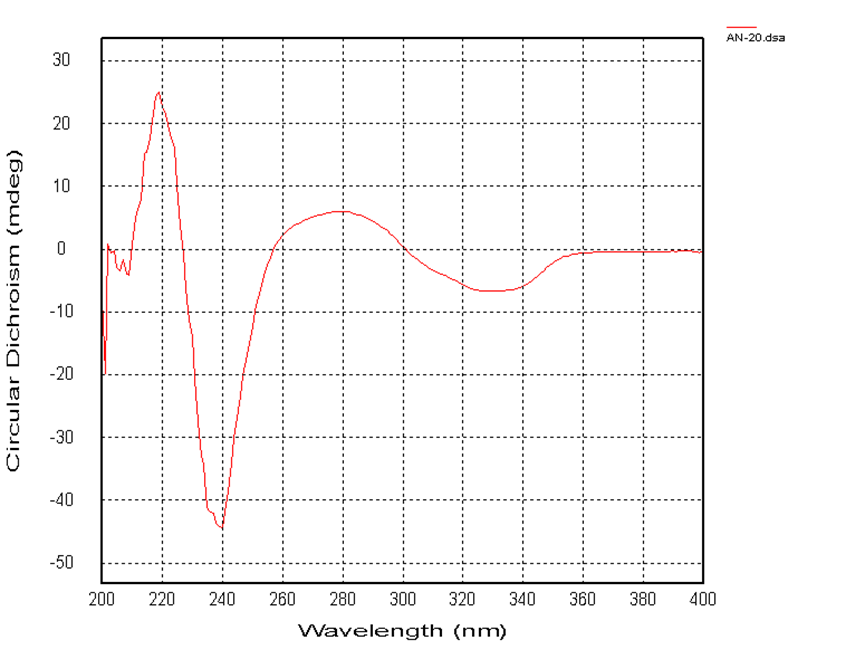


##### Figure S_18_. CD spectrum of 2.

**
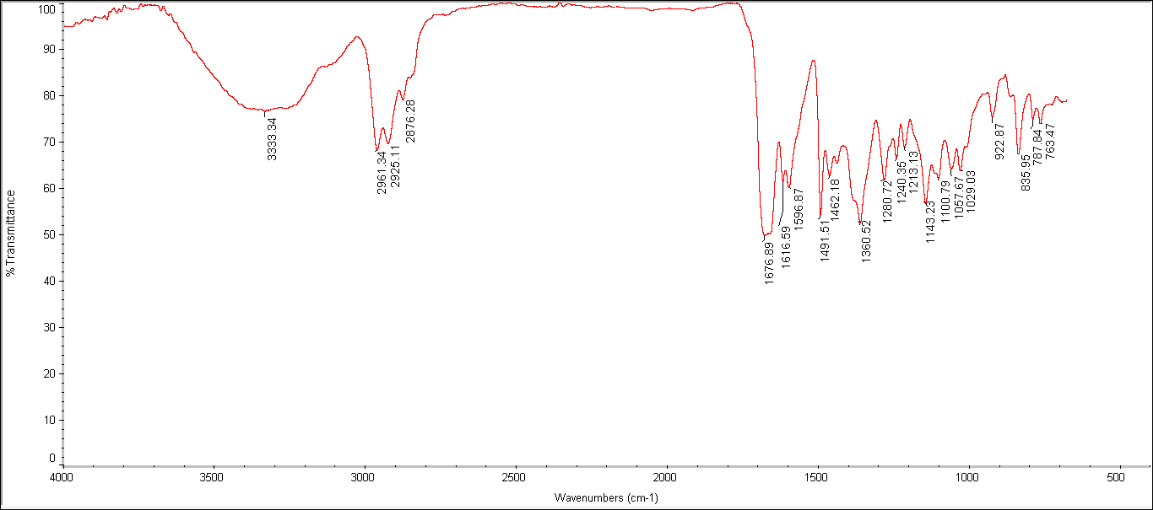
**

##### Figure S_19_. IR spectrum of 2.





**Figure S_20_. Experimental ECD spectra for 2.**
